# Formal Comment on Schorr GS, Falcone EA, Moretti DJ, Andrews RD (2014) First Long-Term Behavioral Records from Cuvier’s Beaked Whales (*Ziphius cavirostris*) Reveal Record-Breaking Dives. PLoS ONE 9(3): e92633. doi:10.1371/journal.pone.0092633

**DOI:** 10.1371/journal.pone.0142287

**Published:** 2015-12-17

**Authors:** Peter L. Tyack, John Calambokidis, Ari Friedlaender, Jeremy Goldbogen, Brandon Southall

**Affiliations:** 1 School of Biology and Sea Mammal Research Unit, Scottish Oceans Institute, University of St Andrews, St Andrews, Scotland, United Kingdom; 2 Cascadia Research, 218 1/2 W 4th Ave., Olympia, Washington, United States of America; 3 Oregon State University Marine Mammal Institute, Hatfield Marine Science Center, 2030 SE Marine Science Drive, Newport, Oregon, United States of America; 4 Department of Biology, Stanford University, 120 Ocean View Boulevard, Pacific Grove, California, United States of America; 5 Southall Environmental Associates Inc., Aptos, California, United States of America; Texas A&M University-Corpus Christi, UNITED STATES

The findings of Schorr et al. [1] involve a poorly understood species that is of significant conservation interest because of its susceptibility to negative impacts from naval mid-frequency active (MFA) sonar systems. While we commend some of the important new data on beaked whale diving presented in Schorr et al. [1], we provide specific comments here because some readers may interpret their findings in ways that may be inappropriate. These include: (1) not adequately appreciating that some of the extreme dives highlighted in the paper were likely response dives; (2) the ways in which the longer-term, lower-resolution tags used by Schorr et al. [1] complement rather than replace the utility of controlled exposure experiments using shorter-term high-resolution archival tags; and (3) that these recent findings do not call into question the response dives documented in DeRuiter et al. [2].

We have concerns about the conclusions of Schorr et al. [1] regarding the “normal behavioral range” of Cuvier’s beaked whales given that some of their extreme “normal” dives may have coincided with exposure to intense sonar nearby. Schorr et al. [1] acknowledge that their data came from an active Navy range and that tagged whales were "almost certainly exposed at some point" to Navy sonar. Falcone and Schorr [3] document many cases in the Schorr et al. [1] dataset where dives from tagged whales occurred near known sonar exercises, but Schorr et al. [1] did not report whether specific dives they highlight as extreme were specifically known to have occurred in relatively close spatio-temporal proximity to ongoing sonar exercises (instead they indicated that analysis of these will be part of a future paper). We think where these relevant contextual factors were known, the authors should have provided what information was available (understandably accompanied by any caveats regarding uncertainty in the data) and we request that they respond to our comment by providing this contextual information at least for the dives that they highlight in their paper as setting depth and duration records.

Schorr et al. [1] discuss how their data are relevant to evaluating behavioral response to sonar and they point out that some of the dives measured in their study were longer than those reported as reactions to sonar in a recent experimental study [2]. However, this comparison must include consideration of whether some of their extreme dives were known to have occurred during sonar exposure and therefore may represent response dives. While Schorr et al. [1] make a point of documenting the longest duration mammalian dive reported to date, they do not report the sonar exposure context of this specific dive. It is important that readers understand that this extreme dive may not represent normal dive behavior, but rather may represent a dive response to sonar exposure. We believe that if this context is known for this, or other dives in their dataset, it is important information that should be presented for interpreting the dive data relative to potential responses to Navy sonar.

Schorr et al. [1] also highlight some of the advantages of the longer term data their tags provide. Because the tags used in their study do not measure sound exposure, Schorr et al. [1] focus on a single tagged whale that traveled farthest from the Navy range to infer effects of a low probability of sonar exposure. This whale had the longest average dive duration in their sample, leading them to “suggest that MFA exposure is unlikely to be a primary factor in the long average dive durations from this dataset” and this whale also had long inter-dive intervals (IDDI), leading them to suggest, “…that factors other than sonar also influence IDDI.” We suggest that conclusions about effects of sonar on diving behavior in the Schorr et al. [1] data set should have waited until the authors complete their analysis of the “subset of this dataset where major sources of acoustic disturbance—or just as importantly, lack thereof–can be accurately documented and independently verified.”

While Schorr et al. [1] accurately tout the tremendous value of their long-term records, it is critical to appreciate that duration of tag attachment is not the only difference between their approach and that of DeRuiter et al. [2]. The short-term, high-resolution archival tags used in experiments reported in [2] to test for responses to simulated sonar sounds were in fact designed for attachment durations well beyond those required to identify the onset of individual responses to short-term controlled sonar exposures. The tags used in [2] sample not only depth and duration of dive, but tri-axial accelerometry and magnetometry, vocal behavior, and received sounds at very high sampling rates. The sensors and sampling rates for these tags were specifically designed to measure sound exposures associated with onset of behavioral responses detected through the multivariate data stream from the tag in ways that are impossible with current satellite tags. Furthermore, DeRuiter et al. [2] explicitly controlled a sonar source to conduct experiments in which the sound exposure at the whale was known and measured with precision. Sound exposure was incrementally increased so that the experimenters could relate any response observed to the sound exposure level that elicited the response. This cannot be directly addressed in uncontrolled conditions, as presented in [1].

Schorr et al. [1] suggest that records as brief as those recorded by short-term tags may lead to sampling errors compared to their longer records. We acknowledge the benefit of long records for many issues, but we do not agree that this point is relevant for interpreting the onset of responses of beaked whales to sonar, which can be identified on a minute-by-minute basis in the high-resolution, multivariate tag data from the controlled exposure experiments. It turns out that some responses of beaked whales to experimental transmissions of sound were so prolonged that the tag fell off before the whales stopped responding [4]. Longer-term records can help to determine how long it takes a beaked whale to return to baseline, but the rich data sets from controlled sonar experiments clearly demonstrate the onset of strong behavioral responses caused by sonar exposure in ways that directly inform regulatory and management decisions. The presence of more extreme dives in the Schorr et al. [1] data should not be taken as undermining the interpretation that the exposure dives reported by [2] represent responses to sonar.

We believe the use of short-term, multi-sensor archival tags with exposures controlled in an experimental design, including the use of sonars on actual naval vessels as has recently begun, represent an important complement to the use of long-term tags with opportunistic Navy sources that Schorr *et al*. [1] report. Each tag type and exposure method has unique advantages and limitations to address elements of sound exposure and response on different spatial and temporal scales and these should not be viewed as competing approaches but rather as valuable complementary tools to address the question of potential responses to sound in different ways.
